# Cheilitis granulomatosa associated with melkersson-rosenthal syndrome

**DOI:** 10.1016/S1808-8694(15)31136-8

**Published:** 2015-10-20

**Authors:** Denise Utsch Gonçalves, Mariana Moreira de Castro, Cláudia Pena Galvão, Alexandre Zoni Rodrigues Brandão, Míriam Cabral Moreira de Castro, José Roberto Lambertucci

**Affiliations:** 1 Medical doctor, ENT specialist, Adjunct Professor of the Ophthalmology, Otorhinolaryngology and Speech Therapy Department of the UFMG Medical School; 2 Medical ENT resident, at the UFMG Clinical Hospital; 3 Medical ENT resident, at the UFMG Clinical Hospital; 4 Medical ENT resident, at the UFMG Clinical Hospital G; 5 Master's degree, medical doctor, ENT specialist. Teacher of the residency program on Otorhinolaryngology at the UFMG Clinical Hospital and the Santa Casa de Misericordia Hospital; 6 Post Doctor, internal medicine specialist, Adjunct Professor of the Internal Medicine Department of the UFMG Medical School

**Keywords:** crohn's disease, cheilitis, melkersson-rosenthal syndrome, treatment

## Abstract

Melkersson-Rosenthal syndrome (MRS) consists of persistent or recurrent orofacial edema, relapsing facial palsy and fissured tongue. The complete triad of symptoms is uncommon, varying from 8 to 25%. The presentation of only one symptom is more common. The most frequent complaint is facial edema and enlargement of the lips. We describe a case of a 17-year-old Brazilian girl with limited edema of the lower lip and fissured tongue due to MRS. Her complaints had started two years before. She referred previous clinical treatments without success. We proposed intralesional injection of triamcinolone at 20 mg every 15 days associated with oral clofazimine at 50 mg/day for three months. The lip became normal after four triamcinolone injections. Recent studies have considered MRS a granulomatous disease, and possibly the initial presentation of Crohn's disease in orofacial area of some patients. MRS patients, therefore, should be screened and monitored for gastrointestinal symptoms. Corticosteroid treatment seems to be effective in reducing lip enlargement. We discus the clinical features of this disease, the treatment, and the importance of corticosteroid therapy in cases of MRS-related facial palsy.

## INTRODUCTION

Swelling of the lips, plicated tongue (lingua plicata), and recurring facial paralysis characterize the Melkersson-Rosenthal syndrome (MRS). The classical triad occurs in 8% to 25% of cases.^1^ Most of these cases present with few symptoms; the usual finding is granulomatous cheilitis (Miesher's cheilitis) which is the most common form.1

Miesher's cheilitis is characterized by recurring and painless localized lip swelling, and is part of the orofacial granulomatosis group (OFG).1 The chronic and recurring nature of the inflammation leads to fibrosis and permanent lip swelling, which makes treatment difficult, and relapses frequent. Histologically it is characterized by non-caseating granulomatous inflammation.^2^, ^3^

Recent studies have suggested an association between the OFG and Crohn's disease (CD), based on the histological similarity between both conditions and the occurrence of MRS manifestations as the initial presentation of CD.^3^, ^4^ Conceivably these findings could be different presentations of the same entity.^5^

The aim of this study is to present the case report of a patient with granulomatous cheilitis and plicated tongue that had an excellent response to treatment with intralesional triamcinolone. We report the clinical progression, treatment options and the possible association between MRS and CD.

## CASE REPORT

A female Caucasian patient aged 17 years presented progressive and persistent swelling of the lips during the last two years.

**Figure 1:**
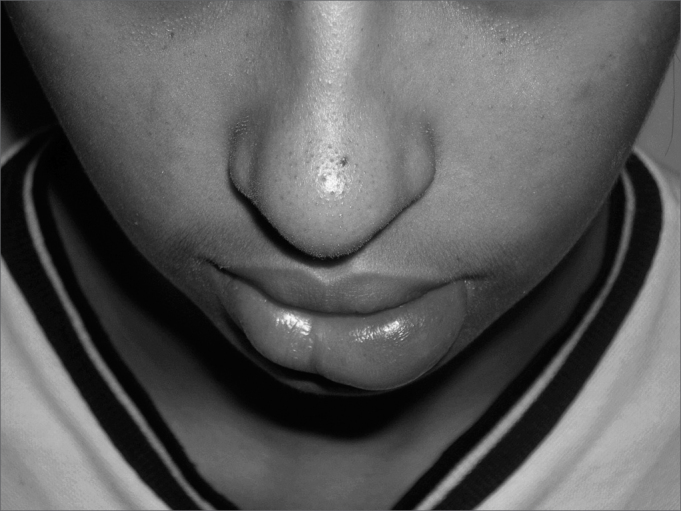
Granulomatous cheilitis with localized swelling in the lower left lip.

The physical examination showed localized swelling in the lower lip (Figure 1) and plicated tongue. Histology of the affected lip revealed inflammation consisting of non-caseating granulomas. An assessment of the digestive tract was within normal limits.

Treatment involved intralesional injection of a corticosteroid (triamcinolone 20mg/ml, 1ml each 15 days during 60 days) and clofazimine (50mg/day) during 90 days. The lip swelling reduced markedly after the first month of treatment. Full resolution occurred after 4 sessions of corticosteroid use.

## DISCUSSION

Orofacial granulomatosis may precede or follow the diagnosis of CD by years. It could be seen as an oral mucosal manifestation of inflammation presenting in the digestive tract.5 Patients with MRS should be warned about the possible development of CD, and should undergo an assessment of the digestive tract.4,5

The accepted treatment of orofacial granulomatosis with lip swelling is the intralesional injection of corticosteroids, combined or not with oral clofazimine.3.^6^ Follow-up shows that local treatment alone is less effective than combined treatment with local corticosteroids and systemic anti-inflammatory drugs.6

## CONCLUSION

The Melkerson-Rosenthal syndrome has been described as a granulomatous disease, which has implications for its treatment. Follow-up of granulomatous cheilitis has shown that intralesional triamcinolone combined with oral clofazimine is an effective treatment.
